# North-Eastern Hospital for Children

**Published:** 1893-06-24

**Authors:** 


### FESTIVAL DINNERS AND MEETINGS.

### NORTH EASTERN HOSPITAL FOR CHILDREN.

The twenty-fifth annual meeting of the Committee of this hospital was held on Wednesday last at Devonshire House, Bishopsgate Street, E.C. The chair was taken by Mr. Lister Godlee. There was a very small attendance, those present being the Lord Mayor, the Rev. W. G. Morcom, Mr. Walter Johnson, Mr. Abram Lyle, and Mrs. Knight. In moving the adoption of the report, the Chairman called attention to the heavy expenses which had been incurred during the past year by the necessity of reflooring one of the wards. Two small legacies had been left to the hospital, and the dinner in aid of the funds had resulted in a sum of £1,200, but he might say that £20,000 was more like the sum needed. The members of the Committee, the president, and vice-presidents having been re-elected, the usual vote of thanks was proposed to the medical staff, secretary, matron, and officers of the institution, Mr. Godlee speaking most warmly of the thoroughly efficient manner in which the work was carried on by one and all, he believed all, without exception, having their hearts in their work. This resolution was seconded by the Lord Mayor. The Rev. W. G. Morcom testified to the good done in the neighbourhood of the North Eastern Hospital by its existence. He himself had never heard one word spoken in its dispraise. The proceedings came to a close with a very cordial vote of thanks to the Chairman, Mr. Godlee being one of the most energetic members of the Committee, and an untiring friend to the hospital.

